# Artificial intelligence, regenerative surgery, robotics? What is realistic for the future of surgery?

**DOI:** 10.1016/j.amsu.2019.04.001

**Published:** 2019-04-17

**Authors:** Sam P. Tarassoli

**Affiliations:** ASiT Medical Student Essay Prize 2019, Swansea University College of Medicine (Year 4), Grove Building, Singleton Park, Swansea, SA2 8PP, UK

**Keywords:** Innovation, Surgery, Medical Technology

## Abstract

The potential of surgery lies in the technological advances that would complement it. The landscape of the field will differ depending on the time period being looked at and would no doubt include conjecture. Initial breakthroughs will need to pave the way for future medical technology and apply to the surgical sciences. Within the next 10 years we would expect to see the emergence of big data analysis, cuttingedge image processing techniques for surgical planning and better implementation of virtual and augmented reality in operating theatres for both patient care and teaching purposes. Over the next 50 to 100 years, the use of quantum computing should lead to increased automation in our healthcare systems. The inception of novel biomaterial invention and advanced genetic engineering will usher in the new age of regenerative medicine in the clinical setting. The future of surgery includes many predictions and promises, but it is apparent that the development will lead to bettering outcome and focus on patient care.

## Highlights

• The landscape of the field will differ depending on the time period being looked at and would no doubt include conjecture.
• Initial breakthroughs will need to pave the way for future medical technology and apply to the surgical sciences.
• In the next 10 years we would expect the use of big data, cutting-edge image processing techniques for surgical planning.
• Over the next 50 to 100 years, the use of quantum computing should lead to increased automation in our healthcare systems.
• Novel biomaterials and advanced genetic engineering will introduce regenerative medicine to the clinical setting.

Modern surgery prides itself on its ability to continually redevelop and renew its techniques and approaches by means of exhaustive trials, studies and peer review. In a discipline shaped by cutting-edge technologies to advance patient outcomes, it would be apt to not only improve current practices, but to also take inspiration from the possible widespread trends of the future. To analyse the potential outlook of surgery one would also need to broadly define ‘future’ in terms of time periods while addressing the realistic possibility of these ideals. The landscape of the field in a decade's time would obviously be different from that same field in 10 decades. Our belief of what the surgical sciences are may be completely unlike what we currently consider it to be.

Much like any technology, there must be the initial scientific breakthrough that paves the way for its development. To have the internet, we needed electricity [1]. To have space travel, we needed jet propulsion [2]. This is also the case in terms of medical breakthroughs. To assess the future of surgery realistically, one must address that there are still challenges to overcome.

The following pages will theorise and postulate the future of surgery in addition to how the field will expect to adapt, develop and shape in the next century. Of course, these are merely concepts based on conjecture, but they are based on trends and the research currently being focused on in the biomedical sciences. One would hope that speculating in such a way could direct where future efforts would.

## 1 The first 10 years

In the last 20 years, we have seen an exponential increase in our computing power and into smaller and smaller form factors. We can even carry in our pocket what was considered a supercomputer 40 years ago. With this development, has come a potential of having such a powerful and portable display; augmented and virtual reality [3]. In the next decade, the use of immersive technologies will become commonplace in and out of the surgical theatre [4,5]. Use of head tracking and motion control sets would allow for greater visualisation both pre-operatively or intra-operatively and with the expansion of network speeds, the ability to carry out procedures remotely could become an ideal of the present [6]. AR and VR not only have a role in direct treatment and management, but also in providing accurate simulation situations to train the next generation of surgeons [7] without compromising their experience or patient care. Additional to this, we should see other uses of our increased computer power in the field of big data [8]. The analysis of large patient populations has the potential to improve outcomes, increase safety and aid service planning [9]. Millions of data sets require our current advancements in data storage [10] to continue and with the emergence of machine learning this can lead to better prediction models for a wealth of diseases. Imaging would also obviously see improvements over the next decade to aid clinician planning (an enhanced image guided system perhaps [11]) or employ cutting-edge processing techniques to more than one image modality to provide information that wasn't accessible beforehand [12]. The next 10 years will not be a case of new sub-specialty industries, but a case of bettering our current technologies and reaching their ultimate potential. Predicting surgical trends further down the line becomes a more difficult task although allows for more creative forecasts of the landscape.

## 2 The next 50 years

In the next half century, there will be the rise of 4 key technologies: artificial intelligence, robotics, genomics and regenerative medicine. Artificial intelligence will build on the machine learning systems we currently have in place [13] to provide surgeons with accurate technical planning and management ability; much like a ‘second opinion’ [14]. It will also allow for rapid analysis of screening data in the preoperative and diagnostic environment [15]. Some have also theorised that artificial intelligence will be the beginning of automation in the surgical field when combined with its ability to learn and adapt to individual patients [16]. Robotic surgery is a blossoming field currently in place in theatres that will be a stalwart of the next few decades [17]. Much like artificial intelligence, the final aim of this technology is to automate procedures [18] to negate human error while keeping a high level of accuracy and precision [19]. Genetic engineering will also play a big part in the next 50 years. The ability to not only rapidly sequence our genes, but also to manipulate this to aid our diagnosis and treatment will be indispensable; for example, targeting defects using CRSIPR-cas9 [20]. It has been predicted that this could introduce a completely new sub-speciality of surgery; genome surgery [21]. Initiatives have been carried out to expand the CRISPR toolbox making gene editing precise, effective and safe [22]. We may see ‘genetic surgeons’ trained for this very purpose. Regenerative medicine incorporates genetic engineering and is the most promising and surgically relevant technology of the future [23]. Its broadly split into the two sectors of tissue engineering and molecular biology. These are combined and used to help repair or replace damaged/unhealthy tissues to normal function. Stem cells, which are a large part of this, are already currently used in some surgical specialties as supplementary treatments [24]. However, their potential lies in the ability to be theoretically manipulated into any cell [25]. Combining this with tissue engineering and 3-dimensional bioprinting and we are edging closer to the possibility of producing bespoke organs. One should not be misled however, as the end goal of a printable kidney is still several decades away, but leaps have been made recently to achieve this [26]. The major hurdle of this technology is not in the availability of raw materials, but in its implementation [27]. We still can't quite get the cells to differentiate and proliferate into what is required and more importantly, we can get them to move to where we want which makes printing a complex organ such as a kidney very challenging. Whether we'll ever have an organ printer in theatre remains to be seen, but the progress that has been made in the last decade has been exponentially quicker than any other current research field [28]. Attempting to predict the future of surgery for the next 50 years is problematic as it is based on current trends. This cannot be applied for forecasts past 50 years as this is purely estimated on broad conjecture.

## 3 To the turn of the century

As discussed previously, initial scientific breakthrough is sometimes needed to occur before another can develop. In the case of space travel, for example, for the ability of interstellar movement with humans to be possible we would need to figure out a propulsion system that can send a ship (with people onboard) past our solar system with the capability of bringing them back to earth [29]. This applies to the future of surgery as well. To become a truly predictive and prognostic science, we need the right tools to achieve it. Despite our computing processing power doubling nearly every 18 months [30], the limit of silicon will be reached, and a plateau met. Eventually mathematical models and problems brought forward by our evolving society cannot be addressed with that processing ability. Quantum computing intends to take the place of silicon computing in this century due to its ability to deal with massive amounts of data with unattainable levels of efficiency [31]. On the spot diagnoses using near instant biochemical and imaging analysis will become a reality [32]. Medical care will be a one-stop establishment for completely personalised service. Simulations of surgical procedures will be run to provide prognostic data and to prepare for any eventuality. This technology can also be used for predictive science; recognising disease burdens and studying large scale populations to apply effective preventative technique [33]. We should also see the arrival of implantable nanotechnology which could carry out internal surgical procedures (without the previous component of invasiveness). Addressing the biomaterials and autonomous features of this technology seem like almost impossible feats as the work is still in its infancy so one can purely speculate at this point.

## 4 The realistic potential

**Fig. 1:**
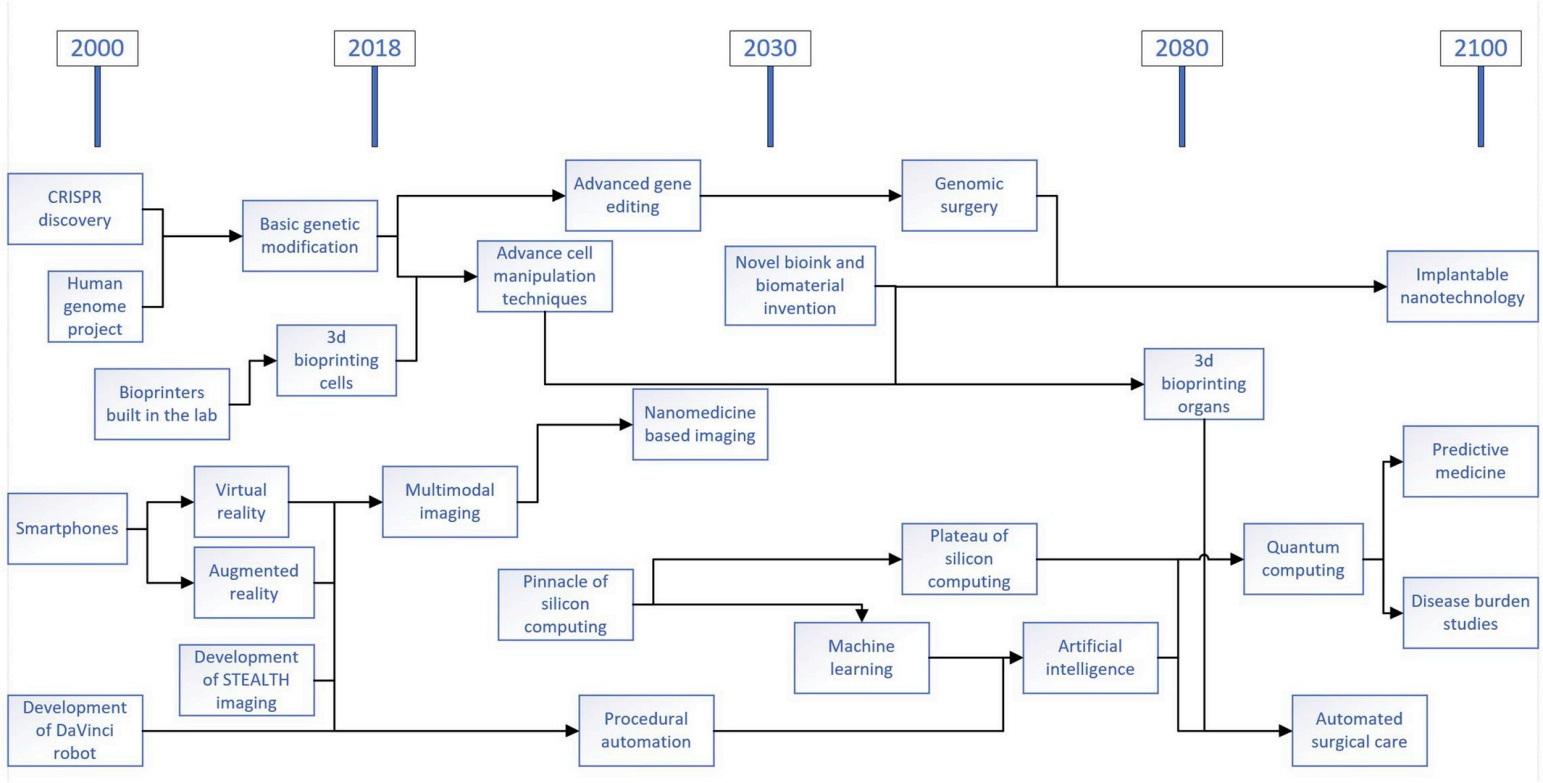
Proposed prediction timeline of future surgical technologies.

One could discuss the future of surgery for as long as time will allow, but without foundations in realism, they would merely be baseless predictions. The next technologies that will be crucial for the betterment of the surgical sciences will either be completely new innovations or an improvement on a current technique or method. As with any industry, developments to equipment and technique will always occur, but the fashion in which they happen depends on the science and technology available to them at that current time. These milestones will hopefully be met in a logical order (Fig. 1), but it's difficult to say if the forecasts would ever stray from this. It should also be noted that these new technologies will not be singular entities for their lifetime, but merge with others.

The future of surgery is full of many promises but also wrought of unknowns. It will depend upon the pace of medical technology development, but more importantly, the field's specific focus on patient care. Fundamentally, these avenues will only find their long place in surgery if they address a specific clinical need while proposing themselves as an improvement to the current technique.

## Ethical approval

N/A.

## Funding

N/A.

## Author contribution

S.P. Tarassoli (Full contribution).

## Conflicts of interest

None.

## Registration unique identifying number (UIN)

N/A.

## RCT, please state the trial registry number – ISRCTN

N/A.

## Guarantor

S.P. Tarassoli.
